# A New Method Combining LDA and PLS for Dimension Reduction

**DOI:** 10.1371/journal.pone.0096944

**Published:** 2014-05-12

**Authors:** Liang Tang, Silong Peng, Yiming Bi, Peng Shan, Xiyuan Hu

**Affiliations:** 1 Institute of Automation, Chinese Academy of Sciences, Beijing, China; 2 Network Information Center, Harbin University of Science and Technology, Harbin, China; Technische Universität Dresden, Medical Faculty, Germany

## Abstract

Linear discriminant analysis (LDA) is a classical statistical approach for dimensionality reduction and classification. In many cases, the projection direction of the classical and extended LDA methods is not considered optimal for special applications. Herein we combine the Partial Least Squares (PLS) method with LDA algorithm, and then propose two improved methods, named LDA-PLS and ex-LDA-PLS, respectively. The LDA-PLS amends the projection direction of LDA by using the information of PLS, while ex-LDA-PLS is an extension of LDA-PLS by combining the result of LDA-PLS and LDA, making the result closer to the optimal direction by an adjusting parameter. Comparative studies are provided between the proposed methods and other traditional dimension reduction methods such as Principal component analysis (PCA), LDA and PLS-LDA on two data sets. Experimental results show that the proposed method can achieve better classification performance.

## Introduction

Dimensionality reduction is a critical pre-processing step in many applications, and several methods have been proposed for dimensionality reduction, such as PCA [1]–[3], Fisher linear discriminant (FLD) [4] and PLS [5], [6]. FLD is sometimes known as LDA and the primary purpose of LDA is to project high-dimensional data onto a low-dimensional space where the data achieves maximum class separability [4], [7]. The derived features in LDA are linear combinations of the original features. The optimal projection or transformation in classical LDA is obtained by maximizing the ratio of between-class and within-class distance, thus achieving maximum discrimination [8]. The intention of PLS is to form components that capture most of the information in the X variables that capture most of the information in the X variables that is useful for predicting ***y***_1_,…,***y*** *_l_*, while reducing the dimensionality of the regression problem by using fewer components than the number of X variables [9]–[11].

In recent years, many approaches have been proposed to overcome the limitations of LDA. The partial least squares discriminant analysis (PLS-DA) using PLS for classification by establishing the connection to Fisher's linear discriminant analysis was properly formalized by Barker and Rayens [5] and Nocairi et al. [12]. This method has been also further developed by Indahl et al. [13] in which an incorporated prior probabilities (associated with the present groups) in the computation of PLS components was suggested. In particular, the existence of high number of irrelevant variables has led to inconsistency of coefficient estimates in the linear regression setting. As a result, Chung et al. [14] proposed sparse partial least squares discriminant analysis (SPLS-DA) which has extended SPLS to solve this problem. By taking advantage of a sequence of data reducing linear transformations (consistent with the computation of ordinary PLS-DA components), Liland et al. [15] developed a powered partial least squares discriminant analysis (PPLS-DA) for computing each component from the transformed data by maximization of a parameterized Rayleigh quotient associated with Fisher's canonical discriminant analysis (FCDA [16]). Telaar [17] introduced an extension of PPLS-DA for optimizing a power parameter towards the final aim, namely towards a minimal classification error. Marigheto [18] proposed a method termed linear discriminant analysis based on partial least-squares (PLS-LDA), where a two-group LDA was performed by using PLS analysis as the reduction step. This method obtained better results than many other widely used classification methods [19]–[22]. Sometimes, small sample problems can be encountered. In this situation, the within-class scatter matrix of LDA is often irreversible. Some researchers have proposed some solutions, for example, using SVD [38] or QR [45] decomposition to solve the within-class scatter matrix. Cai et al. [46] proposed a method called Spectral Regression Linear Discriminant Analysis (SRLDA), which casts the problem of learning an embedding function into a regression framework, by adjusting the regularization parameter, can be avoided to the small sample problem. Schafer and Strimmer [42] proposed a novel shrinkage covariance estimator that exploits the Ledoit-Wolf lemma for analytic calculation of the optimal shrinkage intensity. This shrinkage LDA can guarantee the within-class scatter matrix is always positive definite even for small sample sizes.

Herein we propose two PLS based LDA discriminant methods: LDA-PLS and ex-LDA-PLS. Unlike PLS-LDA in which the PLS method is used for dimension reduction, the proposed LDA-PLS and ex-LDA-PLS uses PLS to adjust the LDA projection direction. The former merely controls the number of PLS latent variables to adjust the LDA projection direction, while the latter combines the LDA-PLS regression coefficient **B** *_lda-pls_* and the LDA projection vector **w** *_lda_* by adjusting a parameter thereby giving more accurate projection direction.

## Background

### Notation

Capital and lowercase letters in boldface denote matrix and vector, respectively. Lowercase italic letters denote the scalars. Matrix dimensions are shown as (*m*×*n*), where *m* and *n* are the number of rows and columns, respectively.

**X** *n*×*k* matrix of samples

**Y** *n*×*m* matrix of sample labels

**Y** *n*×1 vector of sample labels

**B** *_pls_ k*×*m* matrix of PLS regression coefficients

**T** *n*×*a* matrix of the PLS scores for **X**

**P** *k*×*a* matrix of the PLS loadings for **X**

**U** *n*×*a* matrix of the PLS scores for **Y**

**Q** *m*×*a* matrix of the PLS loadings for **Y**

*n* number of samples

*k* number of sample features

*m* number of sample labels

*a* number of components

**S_T_** total class scatter matrix

**S_W_** within-class scatter matrix

**S_B_** between-class scatter matrix

### Overview of Linear Discriminant Analysis

Focused on binary classification problems, LDA finds the set of the most discriminant projection vectors which can map high-dimensional samples onto a low-dimensional space. Using the set of projection vectors determined by LDA as the projection axes, all projected samples will form the maximum between-class scatter and the minimum within-class scatter simultaneously in the projective feature space [23].

Suppose we have two classes of labeled data 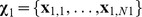 and 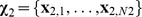. Where 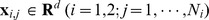 and *d* denotes the sample size of the data dimensionality. Then the centers of two classes are 
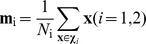
, and two scatter matrices, called within-class and between-class scatter are defined as follows;
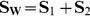
(1.1)where 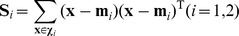




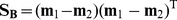
(1.2)The algorithm is as follows.

### Algorithm LDA

**Input:** Two sets of labeled data 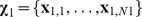 and 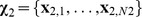


**Output:** Find a linear projection **w** that maximizes the separability between these two classes.

1. Compute the class center of *i*th class 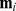 and compute within-class scatter 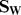 and between-class scatter 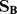

2. Obtain LDA projection by maximizing: 
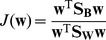

The optimization problem in 
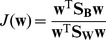
 can be written as follows
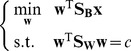
(1.3)


Let 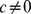, thus we can construct the Lagrangian as;
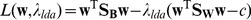
(1.4)

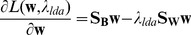
(1.5)


It is clear that 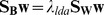, so we can easily obtain the optimal **w** and use the value of new samples after the projection on the **w** as the input parameters of classification [24]–[26]. Herein we will use SVD [38] decomposition to obtain the project vector **w**.

### Overview of Partial Least Squares

Partial least squares (PLS) algorithm was initially suggested by Wold [27]–[29]. It is widely used in social sciences, chemometrics and other fields. The PLS method, which in its classical form is based on the nonlinear iterative partial least squares (NIPALS) algorithm [11]. Many NIPALS based algorithms [47]–[49] are those that incorporate generalized linear regression frame work which is simple and readily feasible to implement. Moreover, it can be easily generalized to any model that is linear at the level of the explanatory variables. The classical NIPALS algorithm is used herein and the following steps are repeated until convergence.

### Algorithm NIPALS

**Input:** Sample data **X** and label matrix **Y**, the number of latent variable of PLS

**Output:**Score matrix of **X** and **Y**, the loading matrix of **X** and **Y**, the regression coefficient matrix **B**.

1. Compute the weight of **X**, 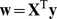, normalize the weight 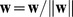

2. Obtain score of **X**, 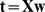

3. Compute the weight of Y, 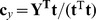, normalize the weight 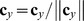

4. Obtain the vector **u**, 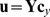

5. Check convergence: compare the **t** with the one from the preceding iteration. If they are equal (within a certain rounding error) go to step 6, else go to step 1
6. Compute the loading vector of **X**, 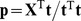

7. Compute the loading vector of **Y**, 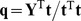

8. Compute the coefficient **b**, 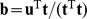

9. Update matrix **X** and **Y**, 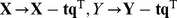

10. Save the vector **t**, **p**, **u, b** and **q**. Back to step 1 and compute the next component

Based on the above description about NIPALS [30], the PLS model tries to determine the multidimensional direction in the **X** space that explains the maximum multidimensional variance direction in the **Y** space, and to decompose the component at the same time on sample matrix **X** and corresponding label matrix **Y**. This is shown in the following formula;
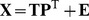
(1.6)

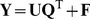
(1.7)

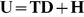
(1.8)where **T** and **U** are score matrices of **X** and **Y**, **P** and **Q** are loading matrices of **X** and **Y**, respectively. The values of **E**, **F** and **H** here are the predicted residuals. Then, we can use the score matrix and loading matrix to do further analysis.

## LDA-PLS Theory and Algorithm

### LDA-PLS Theory

As can be seen from Section 2.2, LDA is a method to obtain the optimal solution **w** such that 
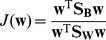
 is maximized, that is, making within-class scatter as small as possible and between-class scatter as large as possible. It differs from the PCA method in that PCA chooses a maximum variance projection direction, while LDA selects the most favorable direction for classification. However, in some cases (Section 3.3), LDA projection direction obtained is not necessarily optimal. Perhaps a slight rotation of the projection direction can achieve the desired direction. A more detailed description of this idea can be found below. Based on this idea, we propose a combination of LDA and PLS dimension reduction method, which we will call LDA-PLS.

The main step of LDA-PLS is that using training data set **X** and corresponding label vector **y** to calculate the LDA projection direction 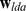 and value 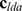 (where 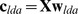 is the projection value of **X** in the 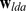 direction), then using the projected value **c** *_lda_* as the label to calculate PLS regression coefficient 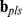, finally subtracting the value in the **w** projection space from **X**, corresponding formula is 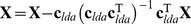. This process is repeated until this meets the number of components we require. The LDA-PLS algorithm can be expressed as (the relevant parameters of PLS optimization object can be found elsewhere [36], [37]);
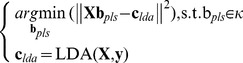
(1.9)where 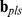 is the regression coefficient of PLS, 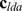 is the projection value of **X** in the LDA direction. 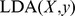 is the LDA algorithm for solving the projection direction, **X** and **y** are input parameters. From the above description, we can easily obtain the LDA-PLS method as follows.

### Algorithm LDA-PLS

**Input:** Sample data **X** and label vector **y**, the number of latent variable of PLS, the component number of LDA-PLS.

**Output:** Coefficient matrix 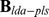 and projection direction 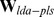 of LDA-PLS.

1. Initial 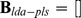, 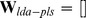

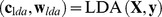

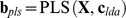

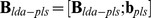
; 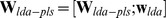

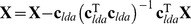

6. Return to step 2 if the iteration number is less than the number of latent variable, else goto step 7
7. Save the coefficient matrix 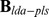 and projection direction 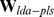

The initial idea is to use 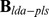 as the model projection direction, thus new samples need only to project on the direction of 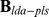. We can use this projected value for classification. The best projection direction may be located between two directions of LDA-PLS corresponding latent variable is 1 and 2. Only by adjusting the latent variable can the optimal direction not be obtained (the best direction is between the red lines with up-triangle and down-triangle). However, the numbers of PLS latent variables are generally integers, conceivable in that such an adjustment is relatively difficult only by latent variables. To have a more fine-tuning capability, we need to assume a combination value between regression coefficient 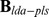 and the LDA-PLS projection matrix 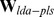. Then we will set the final direction of the model as,
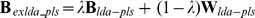
(1.10)


We call this as ex-LDA-PLS algorithm. Here, the *λ* of ex-LDA-PLS is obtained by searching the best value from 0 to 1 and step size is set to 0.001.

### Relationship of the Projections between LDA-PLS and LDA

Here we focus on the relationship between 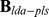 and 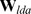, First we briefly recall the formulas related to the PLS regression coefficient **B** (to provide convenience, we will not distinguish 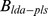 and **B**) such that;
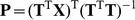
(1.11)

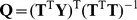
(1.12)

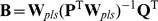
(1.13)


When the number of latent variable is 1, **W_pls_** and **P** are column vectors, **Q** is a constant. Thus Eq. (1.13) can be written as;
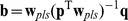
(1.14)


Since **p**, **w** *_pls_* are column vectors, so 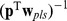 is a constant, q is also a constant, let us set 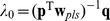. According to the basic PLS principle that 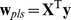, and 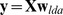 in the LDA algorithm. Using the above Eq. (1.14) gives us;
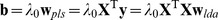
(1.15)


When the number of latent variable is 2, **W** *_pls_*, **P** and **Q** are all matrices, thus we can write the three matrices in the vector form, respectively is 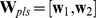, 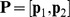 and 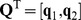. Substituting this into Eq. (1.13) we obtain;
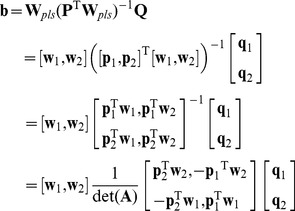
(1.16)where 
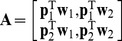
, set 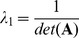, Expanding Eq. (1.16), we can obtain;



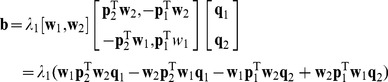
(1.17)Because 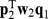, 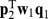, 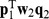 and 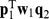 are constant, so we set 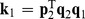, 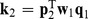, 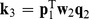 and 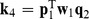. Also we have 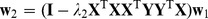 (where 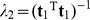 is a constant. Proof can be found in Appendix S1), 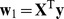 and 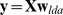. Finally, we obtain;
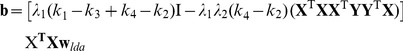
(1.18)


It can be seen from the above expression that LDA-PLS projection 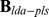 fulfills the requirement of 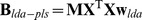, where **M** is a matrix, and **M** changes with different latent variables. This expression indicates the direction **w** *_lda_* of LDA multiplies the left side by matrix 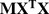 is LDA-PLS projection 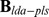. That is, the left multiplication factor is the significance factor of doing the rotation and transformations on **w** *_lda_*. In addition, when the number of latent variables tends to infinity, each 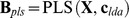 can be fitted consistently, so the LDA-PLS regression coefficient 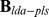 equals to the LDA projection direction **w** *_lda_*.

### Simple Example

**Figure 1:**
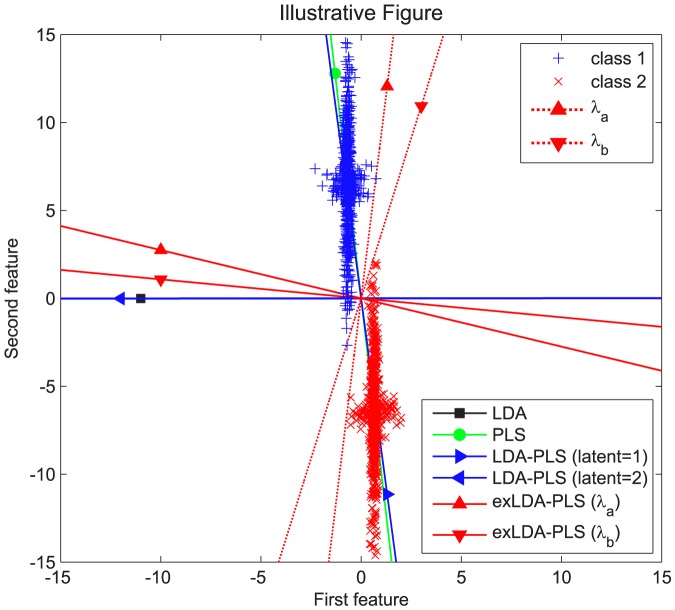
Two dimensional data set with the using of different algorithms. Black line with a square represents the direction of the LDA which coincides exactly with the direction of LDA-PLS when the latent variable is equal to 2 (the proof can be seen from Section 3.2). Red dotted lines of 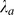 and 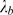 are the boundary lines which can be correctly separate the two kinds of samples, and all lines between these boundaries can also do. Boundary classification 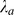 and 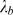 correspond to the direction of ex-LDA-PLS (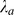) and ex-LDA-PLS (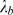).

In order to better understand the LDA-PLS and ex-LDA-PLS algorithm, we construct a special two-dimensional data set, and draw the data set and the projection direction of a variety of algorithms, as shown in Figure 1. In Figure 1, the green line with a circle is the direction of PLS regression coefficient 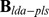, black with square is the direction of the LDA which coincides exactly with the direction of LDA-PLS when the latent variable is equal to 2 (the proof can be seen from Section 3.2). The blue lines with left and right triangles are LDA-PLS projection direction. The red lines with upper and down triangles are the ex-LDA-PLS projection direction achieved by adjusting parameter 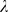, there is often more than one line can be correctly separated the samples in the process to determine the lambda parameter. Here, we only give two boundary lines, ex-LDA-PLS (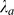) and ex-LDA-PLS (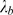). The red dotted lines 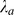 and 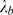 are the classification of ex-LDA-PLS (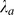) and ex-LDA-PLS (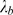) respectively. Optimal classification is given between these two lines. When the center of each kind of samples is determined, then the projection direction of LDA is also determined. If each class does not have horizontal data points, then the LDA direction is the ideal line colored as black in Figure 1. This is a toy dataset with the best classification direction between the two red dotted lines in Figure 1, and the corresponding projection direction in red generated by the ex-LDA-PLS algorithm. The LDA-PLS result is not acceptable currently, primarily because the latent variable uses only integer value 1 or 2. These two latent variables are not able to adjust to the result in the best direction. However the ex-LDA-PLS is readily able to give meaningful results.

## Experimental

### Data Sets

For the purpose of testing and comparing PCA, LDA, PLS, PLS-LDA, shrinkage LDA, LDA-PLS and ex-LDA-PLS methods, we use two data sets. Data set 1 is Raman spectral data, and Data set 2 is a UCI data set consisting Gas Sensor Array Drift (http://archive.ics.uci.edu/ml/datasets.html). Before using the data sets, we removed the non-numerical and missing inputs data, while class label is converted to a numeric type. The details of datasets are shown in Table 1. The Gas dataset was made using an array of 16 metal-oxide gas sensors and a robust gas delivery system [29]. It consisted of a six-gas/analyses classification problem dosed at different concentrations, in which the goal was to discriminate the six different analyses regardless of the concentration. Herein we chose tags for 5 and 6 categories as our classification data.

**Table 1:** Data Sets.

The Name of Data Set	Number of Examples	Number of Attributes	Class label	Year
Gas	4782	128	5 and 6	2012
Raman	925	1834	0 and 4	N/A
Toy	1224	2	−1 and 1	2013

Raman spectral data set were carried out with a micro-Raman setup of a standard Raman spectroscope (HR LabRam Invers, Jobin-Yvon-Horiba). The excitation wavelength of the used laser (Nd: YAG, frequency doubled) was centered at 532 nm. Collectively, there were 2545 spectra for 20 different strains available [31]. Herein we select two classes (*B. subtilis* DSM 10 and *M. luteus* DSM 348).

### Experimental Environment

All programs (http://mda.ia.ac.cn/people/tangliang/code.htm) except shrinkage LDA were performed in house using the Matlab Version R2013a (Math Works, Inc.) and run using a personal computer with a T5550 1.83GHz Intel Core 2 Duo processor, 4 GB RAM, and Windows 7 operating system.

## Results and Discussion

### Estimation of Lambda

**Figure 2:**
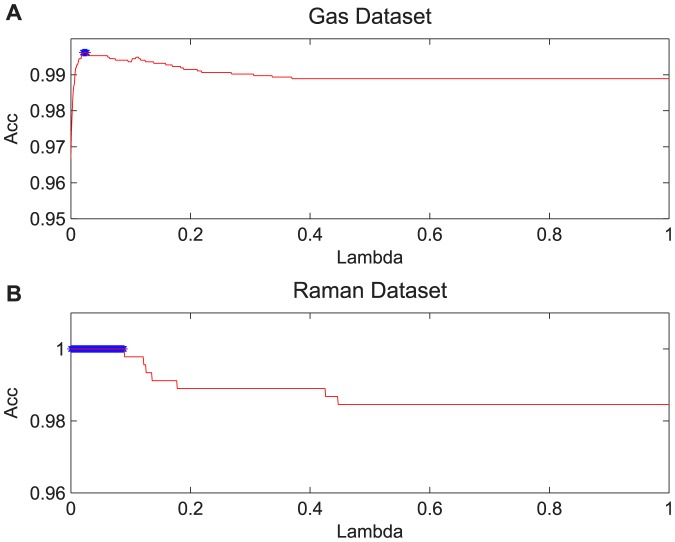
Estimation of Lambda. Acc represent classification accuracy. Red line is the classification accuracy of ex-LDA-PLS algorithm when lambda from 0 to 1, blue line is the best lambda which can make the classification results reach maximum.

For practical application of Eq. (10) one needs to obtain a best estimate 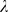. A key question is how to select, one common but also computationally very intensive approach to estimate the 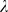 is by using traverse method [39] or cross-validation [40]. Whether traverse method or cross-validation, we can obtain the best lambda only in the training dataset. But the real optimal lambda is to obtain good result in test set, this need the sample is divided evenly. Otherwise, we even can obtain optimal result in the training set. The result in test set often seems not good. As can be seen from Figure 1, the best value of 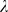 may be a sub-interval between 0 and 1. Our approach is to divide training set into two parts, one is used to select the best 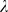, another part is used to verify. Finally, we chose one of the most ideal 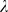 from the validation results. For simplicity, we only took a traversal once to the training set. As can be seen from Figure 2A, the classification accuracy of the best 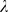 is better than 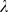 equal to zero (the 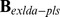 is the LDA projection direction) or one (the 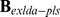 is the LDA-PLS projection direction). In Figure 2B, although found an interval, ex-LDA-PLS can achieve the optimal result, and from Table 2 also can be seen that the accuracy of LDA and ex-LDA-PLS is equal, but Raman is a small sample dataset. The best result in training set may be not necessarily the best in the test set. This phenomenon can be seen from Table 2, the result of ex-LDA-PLS is slightly better than LDA, but less acceptable than LDA-PLS. Theoretically, according to the Eq. (1.10), the result of ex-LDA-PLS will be equal to LDA-PLS or LDA even in the worst case. But this situation is based on having adequate training and test set.

**Table 2:** Classification Accuracy of Different Comparative Algorithms on Raman Dataset.

Algorithm	Train Acc	Test Acc	G-Mean	F-Value
PCA	0.5087	0.4958	0.5259	0.5208
LDA	**1.0000**	0.9603	0.9599	0.9399
PLS	0.9870	0.9841	0.9854	0.9755
PLS-LDA	0.9870	0.9847	0.9861	0.9762
Shrinkage LDA	0.9815	0.9783	0.9815	0.9669
LDA-PLS	0.9935	**0.9848**	0.9849	0.9765
ex-LDA-PLS	**1.0000**	0.9794	0.9758	0.9680

### Comparative Studies on Classification

We conducted a comparative study of PCA, LDA, PLS, PLS-LDA, shrinkage LDA, LDA-PLS and ex-LDA-PLS in terms of classification. The final result contains four indicators, and they were training and test accuracies, G-Mean and F-Value [41]. G-Mean and F-Value are the reasonable evaluation criteria in imbalanced data sets, and they can be simultaneously reflecting the ability to identify the positive samples and negative samples. The algorithm will have more accuracy rate for positive and negative samples when the value of G-Mean and F-Value become higher. In order to evaluate the performance fairly, we used the Bayesian linear classifier [32], [33] for all the above dimension reduction methods. For PLS-LDA, LDA-PLS and ex-LDA-PLS, the optimal variable value for the PLS latent ranged from 1 to 10 was estimated through 10-fold cross-validation in Gas and Raman datasets. Here we will use the cumulative contribution rate [34], [35] assessment strategy for PCA, thus the cumulative contribution rate is set to 0.95. The data was randomly split into training and test sets of ratio 7∶3. The experiment was repeated 20 times. The final accuracy reported is the average of the 20 different runs. The results on Gas dataset are summarized in Table 3. As observed in Table 3, when the cumulative contribution rate is 0.95, the classification result of the PCA seems weaker than the other algorithms. For the LDA method, the result is better than PCA, however less acceptable than others. The primary idea of PLS-LDA is to remove redundancy and noise of high-dimension data set by PLS method, then use the dimension reduction features as the LDA algorithm input, so as to make the PLS-LDA obtain little improvement than PLS in performance. On the basis of LDA, LDA-PLS modifies the LDA projection direction through PLS method. The modified projection direction permitted the dimension reduction features to be more conducive to classification, so as to achieve improved classification result than PLS and LDA methods. Shrinkage LDA achieves better performance, but it is only incrementally less ideal than that of ex-LDA-PLS method. As Schafer suggested [42], the shrinkage LDA will approach the true value in the case of large samples. But after the selection of parameters 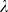, ex-LDA-PLS method obtained better results, and looking at the results from multiple runs, the ex-LDA-PLS was always slightly better than other comparative methods in the cases of large samples. Because of extensive collinearity of neighboring features in Raman spectra dataset [43], [44], PCA algorithm can only obtain one eigenvector when the cumulative contribution rate is 0.95, and the results were often unacceptable. Raman dataset can be regarded as a small sample set, the number of features larger than the number of samples. In this case, LDA with eigenvalue decomposition cannot compute the inverse of within-class scatter matrix. Here we use SVD for solving the inverse matrix, and as can be seen from Table 2, the overall performance of LDA is acceptable. The algorithms which based on PLS method can solve small sample size problem, the results of PLS and PLS-LDA are somewhat the same, better than shrinkage LDA and ex-LDA-PLS. From the results, although the LDA result slightly less ideal, but after adjustment by PLS, the LDA-PLS result was effectively equal to PLS and PLS-LDA. Since Raman is a small dataset, even a projection direction can be completely separate all the training samples, on the test set is not necessarily the best, this depending on the overall distribution of the sample. Herein, the ex-LDA-PLS result is no more than LDA-PLS.

**Table 3:** Classification Accuracy of Different Comparative Algorithms on Gas Dataset.

Algorithm	Train Acc	Test Acc	G-Mean	F-Value
PCA	0.7896	0.7902	0.8103	0.7976
LDA	0.9652	0.9576	0.9615	0.9646
PLS	0.9869	0.9842	0.9870	0.9869
PLS-LDA	0.9866	0.9848	0.9875	0.9874
Shrinkage LDA	0.9934	0.9912	0.9928	0.9928
LDA-PLS	0.9912	0.9896	0.9913	0.9914
ex-LDA-PLS	**0.9962**	**0.9922**	0.9934	0.9936

### Recognition rate vs. number of components

**Figure 3:**
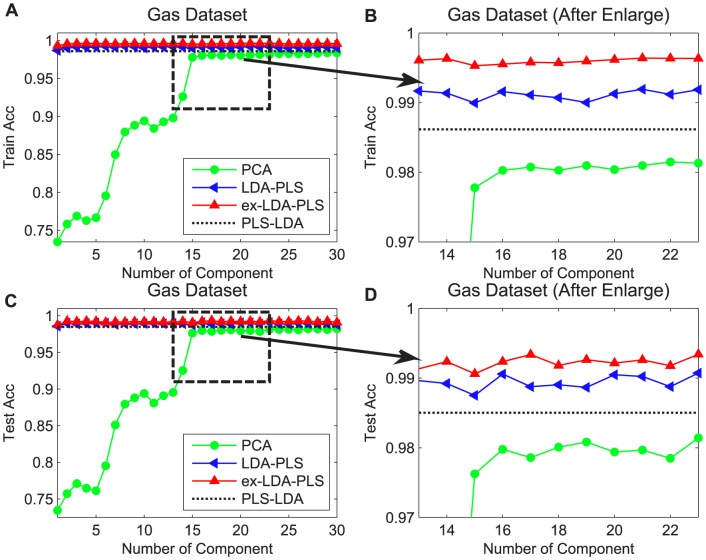
Recognition rates vs. number of components on PCA, LDA-PLS, ex-LDA-PLS and PLS-LDA in Gas dataset. Acc represent classification accuracy. Green lines with circle represent the results of PCA, blue lines with left triangle are LDA-PLS classification results, red lines with up triangleare the ex-LDA-PLS results, and black dot lines are the mean result of PLS-LDA.

**Figure 4:**
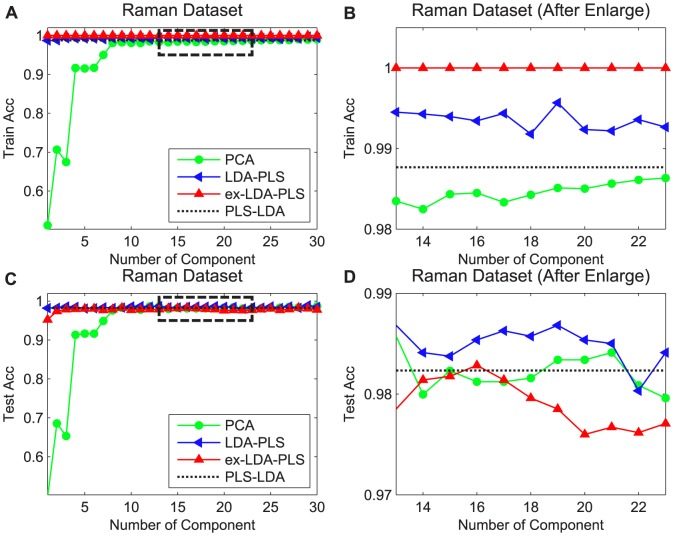
Recognition rates vs. number of components on PCA, LDA-PLS, ex-LDA-PLS and PLS-LDA in Raman dataset. Acc represent classification accuracy. Green lines with circle represent the results of PCA, blue lines with left triangle are LDA-PLS classification results, red lines with up triangle are the ex-LDA-PLS results, and black dot lines are the mean result of PLS-LDA.

Herein we show how the average recognition rates of the LDA-PLS, ex-LDA-PLS, PLS-LDA and PCA algorithms change depending on the number of extracted components. The fundamental condition of experiment is similar to the above, in that each principal component reported here use the final accuracy of an average of the 20 different runs. The difference is the numbers of principal components of PCA which are not based on the cumulative contribution rate for the standard, but from 1 to 30. The LDA-PLS and ex-LDA-PLS algorithms use the same strategy for components. Because there was only one component after executing PLS-LDA algorithm, we assumed the mean values as PLS-LDA results, as shown in the graph as dot lines. The latent variables of Raman and Gas data set are set to optimal values by cross validation method, Solution λ in ex-LDA-PLS is analogous as described in Section 5.1. It is clear from Figure 3 that the classification accuracy of PCA increases with the number of principal components, and tends to be stable when reaching a certain value. While the LDA-PLS and ex-LDA-PLS method is less sensitive to the number of components, thereby fundamentally maintaining a relatively stable value. The primary reason for this situation is that the remaining value of **X** after several times iteration contains little information, so even if there are more components, only a few have a dominant role. In gas dataset, the number of LDA-PLS and ex-LDA-PLS components can achieve good results assuming the value is less than five, while PCA needs to assume 15 principal components to obtain better results than LDA-PLS. To reach the similar results for the ex-LDA-PLS, of course we can also assume a larger value to increase the classification accuracy than that of LDA-PLS, in that we cannot achieve the effect of dimension reduction. For the Gas dataset, classification accuracy increased with the increase of the cumulative contribution rate. When the cumulative contribution rate is 0.99 (the number of principal components for almost 15 or so), the PCA method achieved similar performance with PLS-LDA. However the number of components was much larger than the other three methods. Figure 3B is an enlarged result of the black box in Figure 3A, and Figure 3D is the enlarged results of Figure 3C. From the Figure 3B and 3D can be seen that the results of ex-LDA-PLS maintain consistency and are better than LDA-PLS in the training and test set. The primary reason is that Gas is a large sample dataset, the founded direction in the training set is also appropriate to test set. As for Figure 4A and 4C in Raman data set, the LDA-PLS can achieve better results. In order to obtain somewhat the same results with LDA-PLS and ex-LDA-PLS, we need to assume that the PCA principal components number to 10 or so. From the perspective of the cumulative contribution rate, in the Raman data set we found that the classification result still cannot be compared with other the three methods even though the cumulative contribution rate are 0.98,0.99,0.999 (0.999 corresponding to the number of principal components with the value of between 1 to 2). As can be seen from Figure 4B and 4D, ex-LDA-PLS achieved consistent results which were better than LDA-PLS and PCA in the training set. However this became unacceptable as compared to than LDA-PLS and PCA, the primary reason for this phenomenon was that Raman was a small sample set. The distribution of the training set is not representative of the entire samples, so even ex-LDA-PLS can obtain the best direction in training set, the test set not being optimal. This shows that ex-LDA-PLS is not easy to obtain the best results in the small sample dataset.

### Impact of PLS Latent Variables

**Figure 5:**
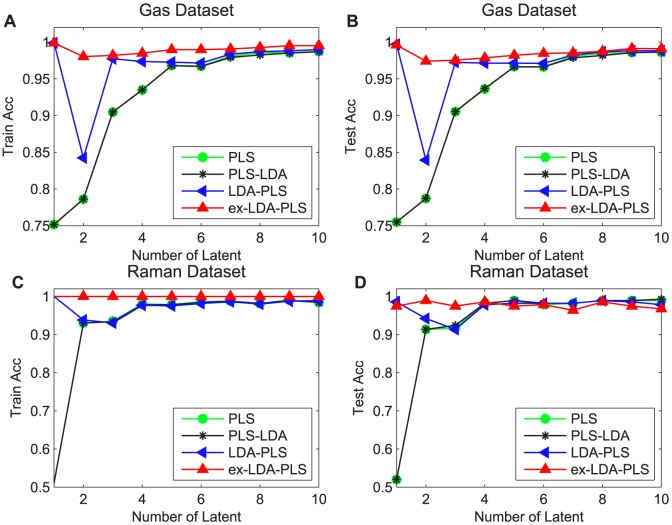
Relationship between the number of PLS latent variables, PLS-LDA, LDA-PLS and ex-LDA-PLS algorithm performance. Red lines with up triangle represent the results of ex-LDA-PLS, blue lines with left triangle are LDA-PLS classification results, green lines with circle are the PLS-LDA results, cyan lines with star are the PLS results. Square and star represent the training set and testing set, respectively.

In the experiment we assumed the maximum number of latent variables to be equal to the features of the dataset and for each latent. We also report the final accuracy by the average of the 20 different runs. The abscissa in Figure 5 represents the number of latent variables and **y** coordinates for the classification accuracy. Since LDA method has not latent variable parameter, we show that the classification results are corresponding to each latent variable of PLS, PLS-LDA, LDA-PLS and ex-LDA-PLS. In order to facilitate observation, we draw the classification accuracy of the train set and test set. It can be obtained from the front theory that the primary idea of LDA-PLS is to multiply a matrix **X** ^T^ **X** on the left side of the original LDA projection direction when the latent variable is 1. When the latent variable is greater than or equal to 2, then the projection direction of LDA-PLS equivalent to the direction of LDA left multiplied by a matrix **MX** ^T^ **X** (where **M** is a matrix, can be seen from Section 3.2). The ex-LDA-PLS is based on LDA-PLS and LDA so as to emphasize the projection direction which is more consistent with the actual situation. In theory, when the number of latent variables tends to infinity, LDA-PLS, ex-LDA-PLS and PLS projection direction should coincide in that it is manifested in the experimental results with the classification accuracy tending to be equal. In Figure 5, the two data sets have almost the same trend. When the latent variable values were relatively small, the classification accuracies of LDA-PLS and ex-LDA-PLS were significantly higher than the PLS and PLS-LDA method. While the latent variable value reached a specific value, the results of LDA-PLS, ex-LDA-PLS and PLS tended to be as the same. In this experiment, the PLS latent variables of the two data sets were controlled to within 10.

## Conclusions

Herein we have presented a new dimension reduction method based on PLS and the traditional LDA, by adjusting the number of PLS latent variables to change the projection direction of the LDA. In some cases, the change can make projection more conducive to the classification results. Our future work will include an in-depth analysis of the scope of LDA-PLS and ex-LDA-PLS algorithms and to solve multi-classification problems while considering the joining of kernel ideas to solve nonlinear problem.

## Supporting Information

Appendix S1 **The relationship between w_1_ and w_2_.**
(DOCX)
