# Supplementary material for: A New Method Combining LDA and PLS for Dimension Reduction
Source: PLoS One. 2014 May 12;9(5):e96944. doi: 10.1371/journal.pone.0096944 (PMC4018361; doi:10.1371/journal.pone.0096944)
Supplement: Appendix S1 — The relationship between w1 and w2. (DOCX) [file pone.0096944.s001.docx]

The NIPALS algorithm based on Section 2.3, we can write the three matrix elements in the vector form, respectively is$W_{pls}=\left[w_{1},w_{2}\right]$,$P=\left[p_{1},p_{2}\right]$and$Q ^{T}=\left[q_{1},q_{2}\right]$when the latent variable is 2.

At the time of iteration to the second step, using Eq. (1.20) and Eq. (1.21) into$w_{2}=X_{2}^{T}y_{2}$gives;

$$w_{2}=X_{2}^{T}y_{2}=(X-t_{1}p_{1}^{T})^{T}(y-t_{1}q_{1}^{T})$$

Expand Eq. (1.23) and use$p_{1}=X^{T}t_{1} / (t_{1}^{T}t_{1})$, $q_{1}=y^{T}t_{1} / (t_{1}^{T}t_{1})$into it, we will obtain;

$$\begin{matrix} w_{2}=X_{2}^{T}y_{2} \\ =(X - t_{1}p_{1}^{T})^{T}(y - t_{1}q_{1}^{T}) \\ =X^{T}y - X^{T}t_{1}q_{1}^{T} - p_{1}t_{1}^{T}y + p_{1}t_{1}^{T}t_{1}q_{1}^{T} \\ =w_{1} - X^{T}t_{1}(t_{1}^{T}t_{1})^{-1}t_{1}^{T}y - X^{T}t_{1}(t_{1}^{T}t_{1})^{-1}t_{1}^{T}y + X^{T}t_{1}(t_{1}^{T}t_{1})^{-1}t_{1}^{T}t_{1}(t_{1}^{T}t_{1})^{-1}t_{1}^{T}y \\ =w_{1} - X^{T}t_{1}(t_{1}^{T}t_{1})^{-1}t_{1}^{T}y \end{matrix}$$

Because$t_{1}$is a column vector, so$(t_{1}^{T}t_{1})^{-1}$is a constant variable, we set$\lambda_{3}=(t_{1}^{T}t_{1})^{-1}$, joint Eq. (1.22) to obtain;

$$\begin{matrix} w_{2}=w_{1} - \lambda_{3}X^{T}t_{1}t_{1}^{T}y \\ =w_{1} - \lambda_{3}X^{T}XX^{T}yy^{T}XX^{T}y \\ =w_{1} - \lambda_{3}X^{T}XX^{T}yy^{T}Xw_{1} \\ =(I-\lambda_{3}X^{T}XX^{T}yy^{T}X)w_{1} \end{matrix}$$
